# Vienna Letter

**Published:** 1890-02

**Authors:** Hugh Hagan

**Affiliations:** Vienna, Austria


### VIENNA LETTER.

---

VIENNA, AUSTRIA, Dec. 23, 1889.

During our rounds in the insane asylum, under Professor Krafft Ebing, I have noticed very little that differed materially from similar institutions with us. I was much impressed with an isolation bed they used to a great extent, and which is highly recommended by Professor Ebing. This makes an admirable cage, so to speak, and for acute maniacal patients, epileptics, general paralytics, and in whatever case a nurse be needed, of course the most violent excepted, the bed plays a dual part of nurse and isolated room, for it is impossible for the patient to do himself an injury or to fall out of the bed. Every ward has three or four such beds.

Vienna has been smitten with an epidemic of the so-called Russian influenza. The weather has been peculiarly suited for such an invasion, for there have been daily, snows, sleets, and cold drizzling rains for three weeks, and not a ray of sunshine.

Almost every man, woman and child have had the influenza, and there has been considerable talk over the subject, in the medical world.

The disease has a stage of incubation of about two to three days, accompanied with a feeling of malaise. Then the stage of invasion, which varies from a few hours to a day or two, is quite severe—being ushered in by severe rigors and chilly sensations.

Then by a high temperature ranging from 102 degrees to 104 degrees F., with terrible headache, pains in limbs, joints, between the shoulders; nausea, vomiting, and a feeling of fullness in the eyes.

There may be even slight delerium, and I have seen several cases where the nervous symptoms were most pronounced. The symptoms are grave enough to cause great anxiety, but fortunately after the second or third day a rapid convalescence ensues. There have been no fatal cases reported.

A specific organism is believed to be the cause, but as yet, has not been isolated. The only pathological change known, is a severe congestion of the nasal mucous membrane.

The prognosis is without exception favorable. The treatment pursued by Viennese physicians is either to let it run its course or combat it. With an active purge of calomel, or a saline cathartic, opium or the bromides for the pain and restlessness, and a low diet. We might give a gramme or so of sulfonal, if desired, to allay any possible sleeplessness.

I have been endeavoring to see, to what extent Charcot's theory of suspension in locomotor ataxia, was used in Vienna, but I find, without exception, that the whole faculty are opposed to it, on two very important points; first they have seen no permanent good result from the practice, and secondly, that six or eight fatal accidents have come to light, even though the suspension was performed by experienced physicians.

Professor Nothnagle has invented an extension jacket, something after Dr. Sayre's plaster jacket, only in Prof. Nothnagle's jacket, there are two halves, a superior and an inferior connected by an extension screw, back and front, the points of rest are the crests of the ilium and the axillæ.

I have never seen the jacket tried, and can find no advocate for its use.

Sulfonal is used in the general hospital and in the insane asylum to such an extent that one looks on in wonder. It has almost entirely taken the place of chloral, the bromides and paraldebyœ, the latter being tried after sulfonal.

The doses given are generally large; from 15, 20, and 30 grains at a dose, and repeated in two hours, if sleep be not produced. Milk is the medium prefered, a glass is given with the dose of sulfonal.

For insomnia, whether of melancholic, maniacal, anæmic, and what not, sulfonal, in 15 to 30 grain doses, will be found satisfactory. The sleep produced is refreshing, and of about six to eight hours' duration. No bad results have been noted from these large and frequent doses.

HUGH HAGAN, M. D.

---

QUINSY.—In the early stages of quinsy, chloral hydrate is nearly a specific, three or four grains to the ounce of glycerine being used as a gargle. It is locally antiseptic, astringent and sedative.—*Medical Record.*

